# Anti-NMDAR limbic encephalitis- a clinical curiosity

**DOI:** 10.1186/1477-7819-12-256

**Published:** 2014-08-09

**Authors:** Abhay K Kattepur, Darshan Patil, Amarendra Shankarappa, Shivananda Swamy, Nayakanur Shankarappa Chandrashekar, Pushpa Chandrashekar, Shailesh Prabhu, Kodaganur Srinivas Gopinath

**Affiliations:** Department of Surgical Oncology, Anesthesia* and Critical Care, HCG Bangalore Institute of Oncology, No 44-45/2, 2nd cross, Bangalore, 560027 Karnataka India

**Keywords:** Paraneoplastic, Limbic encephalitis, Anti-NMDA, Ovarian teratoma

## Abstract

**Background:**

Neurological paraneoplastic syndromes are rarely the first manifestation of an underlying cancer. A high index of suspicion is thus needed to diagnose such conditions. Paraneoplastic limbic encephalitis is one such entity which is well described in association with small cell lung cancers, testicular germ cell tumors, breast cancers and ovarian tumors. This article describes the entity being associated with an ovarian tumor.

**Case:**

A 36-year-old female presented with abnormal behaviour, mood swings and delusions. She was evaluated for her psychiatric symptoms and found to have an underlying ovarian tumor. Anti-NMDA receptor titers were strongly positive. She underwent oophorectomy, and post-operatively there was a significant improvement in her psychiatric symptoms.

**Conclusions:**

Ovarian tumors like teratomas are implicated in the pathogenesis of paraneoplastic limbic encephalitis. An underlying ovarian tumor must be evaluated in all young females presenting with sudden onset of psychiatric symptoms.

## Background

Paraneoplastic limbic encephalitis (PLE) was first described by Corsellis *et al.* [1]. This entity was initially described in association with brainstem dysfunction affecting older individuals with lung cancer. Subsequent studies, however, have defined PLE in association with germ-cell tumors with dominant limbic, diencephalic and upper brainstem dysfunction [2]. Anti-N-methyl-D-aspartic (NMDA) receptor (NMDAR) encephalitis is one such acute, and a potentially lethal, form of limbic encephalitis. It was described as a distinct disease entity by Dalmau *et al.* in 2006. More than half of the cases described have a paraneoplastic association with tumors, especially ovarian teratomas [3]. The frequency of this disorder is largely unknown [4].

## Case presentation

A 36-year-old married female with no comorbidities presented with a history of abnormal behaviour of five weeks which included mood liability (laughing/crying spells), delusions of persecution (feeling that other people were trying to harm her), irritability and occasions of talking to herself. She also had urinary and fecal incontinence. For these complaints, she was shown to a psychiatrist, who evaluated her and started her on anti-psychotic drugs. However, even with adequate doses of medications, the symptoms worsened. An organic brain pathology was therefore suspected, and she was referred to a neurologist. Blood tests, which included tests for serum electrolytes, viral markers, thyroid profile and anti-phospholipid antibodies (APLA), anti-nuclear antibodies (ANA) and anti-nuclear cytoplasmic antibodies (ANCA) were normal. A magnetic resonance imaging (MRI) brain scan and an electroencephalogram (EEG) were also normal. However, anti-NMDA antibody titers were strongly positive.

**Figure 1:**
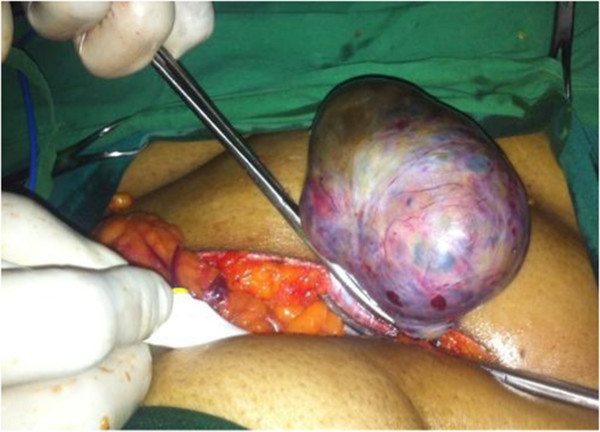
**Intra-operative photograph.** The tumor arising from the right ovary.

**Figure 2:**
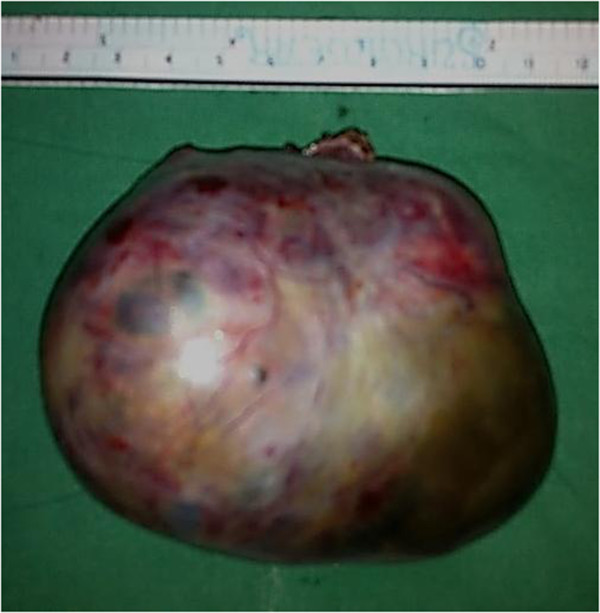
**Specimen photograph.** The excised right ovarian tumor, measuring 8.5 cm.

**Figure 3:**
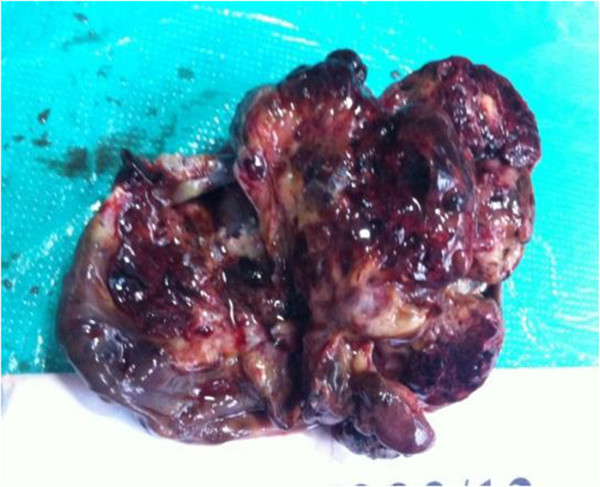
**Specimen photograph (cut section).** Grey-white tumor with cystic areas.

**Figure 4:**
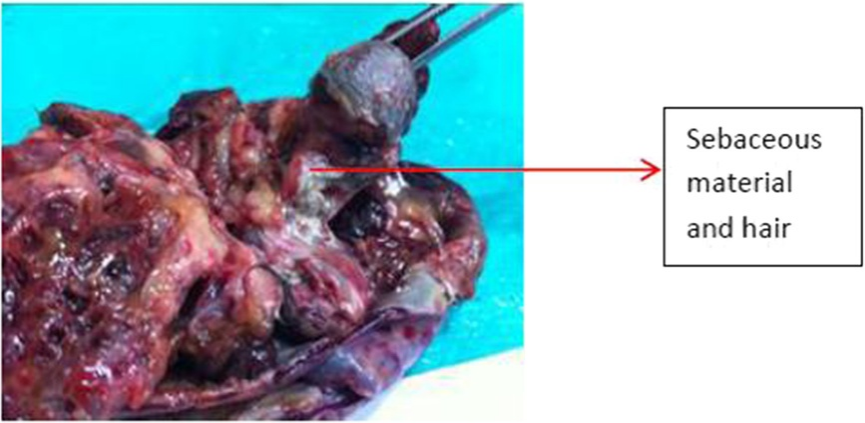
**Specimen photograph (cut section).** Sebaceous material and tufts of hair (arrow) are seen on closer inspection.

Thus, a diagnosis of anti-NMDA autoimmune encephalitis was made. She was started on steroids at a dose of 1 g/day. However, symptomatic improvement was minimal. A paraneoplastic association was suspected, and a computerized tomography (CT) scan of her abdomen was done which showed a 8.3 × 6.9 × 5.3 cm heterodense lesion arising from her right ovary with calcification suggestive of a dermoid cyst. She was then referred to us for surgical management of the adnexal lesion. On examination, our patient was fidgety and restless with poor word output. There was no eye-to-eye contact during conversations. A mini mental status examination (MMSE) showed a score of 4 out of 30. She was not even able to walk or to write legibly. Our patient underwent a right oophorectomy under general anesthesia (Figure 1). The tumor grossly measured 8.5 cm in the largest dimension (Figure 2). On sectioning, sebaceous material and hair were seen (Figures 3 and 4), suggesting an ovarian teratoma.

Post-operatively, our patient was on a ventilator for two days. Later she was transferred to ward and managed with anti-epileptics and steroids. By the end of the first week, she was able to talk with her family members and ask for food and water; by the 10th day, she was making eye-to-eye contact during conversations with good word output; by the 20th day, she was able to climb four flights of stairs and write legibly; by the end of one month, she was doing her household chores and office work; her MMSE score had improved to 26 out of 30.

## Discussion

### Pathophysiology

Anti-NMDAR encephalitis is mediated by auto-antibodies against the NR1/NR2 subunits of the receptor [5]. NMDA receptors are heterodimers of NR1 and NR2 subunits, with both subunits being required to create a functional receptor [4]. Physiologically, the NMDA receptor is important for synaptic plasticity and, in turn, is responsible for higher functions such as learning and memory [5].

The pathogenesis involves autoimmune over expression of NR2 subunits by nerve tissues in the teratomas that lead to a break in the immune tolerance. Associated factors such as a prodromal viral-like illness and genetic factors may play additional roles in the initiation of an immune response [4]. Inhibition of NMDA receptors by these auto-antibodies leads to a reduced gamma-aminobutyric acid (GABA) release in presynaptic neurons that in turn switches off the inhibitory effects of glutamate release in the postsynaptic neurons of the prefrontal or subcortical areas, thereby contributing to the development of psychosis and dyskinesias. The clinical manifestations vary according to the receptor subtype to which the antibodies bind. Binding to the NR1 subunit leads to hypoventilation, while binding to the NR2A subunit leads to amnesia.

About 60% of patients with PLE have an underlying tumour. As stated earlier, most commonly the cause is an ovarian teratoma, but tumours such as testicular teratomas and small cell lung cancers have also been associated with this entity [5]. Tumours occur in about 59% of all cases of anti-NMDAR encephalitis [6].

### Clinical features

This entity has been typically described: 18 years to 30 years female patients present with a characteristic neuropsychiatric syndrome of personality and behavioral changes, paranoia and memory disturbances [3]. Such patients are often mistaken for psychiatric patients and are often admitted to psychiatric centers [4]. Later during the course of the illness, multiple neurological deficits, dystonias, dyskinesias, seizures, autonomic instability and clouding of consciousness occur [3, 4]. Orofacial dyskinesias (jaw opening and closing, chewing, teeth clenching, facial grimacing, lip pouting) and tongue protrusion are characteristic features of this illness [5]. Once seizures are controlled, any attempts to decrease the sedation or wean patients off the ventilator lead to are appearance or worsening of the abnormal movements [4]. Central hypoventilation can occur in those with brainstem and/or hypothalamus involvement.

Iizuka *et al.* have grouped the symptoms of anti-NMDAR encephalitis into five characteristic phases: prodromal, psychotic, unresponsive, hyperkinetic and gradual recovery [5]. Clinical recognition of this syndrome is important for two reasons: 1) the sub-acute presentation in a young woman often leads to an initial diagnosis of acute psychosis, malingering or drug abuse, and 2) despite the severity of symptoms, patients usually recover [2].

In our case, the patient had some of the above-mentioned features in addition to bowel and bladder incontinence.

### Diagnosis

Diagnosis is often difficult, requiring certain tests to rule out other causes of encephalitis. These include a cerebrospinal fluid (CSF) examination that shows pleocytosis or increased protein concentration, suggesting an inflammatory or immune-mediated neurological process [4]. A MRI scan is often unrevealing; although some patients (55%) [3, 5] have increased signals on FLAIR or T2W images in the medial temporal lobes followed by the cerebral cortices, cerebellum, brainstem and basal ganglion,these findings are non-specific and correlate poorly with symptoms. Abnormalities in EEGs are seen in only 21% of patients, the usual finding being generalized or predominantly fronto-temporal slow waves [5]. Recently, a unique EEG pattern has been identified in this condition called ‘extreme delta brush’, which is a pattern of waveforms resembling those seen in premature babies and this pattern is associated with a prolonged illness [7]. The definitive diagnosis is based on NMDAR antibodies identified in serum or CSF [3]. Antibody titres in CSF are usually higher than in the serum and correlate well with clinical symptoms. No false positive results have been reported to date [5].

In our case, the CSF showed pleocytosis, the EEG was abnormal, the MRI scan was normal and antibody titers to NMDAR were high.

Thus, a diagnosis of PLE is made based on the following criteria [8]:

1. Compatible clinical picture.
2. An interval of < 4 years between the development of neurological symptoms and tumour diagnosis.
3. Exclusion of other neuro-oncological complications.
4. At least one of the following: CSF with inflammatory changes but negative cytology; MRI demonstrating temporal lobe abnormalities; EEG showing epileptic activity in the temporal lobes.

### Differential diagnosis

An important diagnosis that should be kept in mind and excluded is herpes simplex encephalitis [9]. This often presents with cognitive changes, seizures, tremors, myoclonus and stroke-like symptoms and is characterized by a relapsing course. Other entities included in the differential diagnosis are prion diseases, autoimmune vasculitides, systemic autoimmune diseases and steroid-responsive encephalopathy associated with autoimmune thyroiditis. The wide range of abnormal movements and prominent orofacial involvement that remain even when the conscious level is low, and the resistance to treatment, together with psychiatric symptoms, autonomic features and central hypoventilation, distinguish anti-NMDAR encephalitis from other types of paraneoplastic encephalitis [5].

### Treatment

The treatment of PLE includes corticosteroids, intravenous IgG (IVIG) and occasionally plasma exchange [9] along with antiepileptic medications, sedation, mechanical ventilation, nutritional support and management of episodes of autonomic instability and dyskinesias. Some patients have transient (usually partial) improvement; such patients are often discharged without a final diagnosis and they are the subset of patients who subsequently deteriorate or succumb if the ovarian tumor has not been removed [4]. Early recognition and treatment appears to be critical to achieve a favorable outcome. Also one should not wait for a positive NMDAR antibody result, which can take up to several weeks to be processed. The importance of initiating aggressive immunomodulatory therapy even without evidence of a tumour cannot be overemphasized, and it strongly argues against the use of empiric ovarian resections, which has been advocated by a few [3]. Surgical treatment is straightforward and involves removal of the ovarian tumour. Since most of the patients are young adult women, preservation of reproductive function should be the goal. During surgery, propofol is a more appropriate anaestheticto be used for such patients. On the contrary, volatile anesthetics may suppress the immune response more potently compared with propofol and must be avoided [6]. Recovery during the post-operative period is often slow, and weaning of the patient off the ventilator must be judiciously interpreted with clinical parameters. However, some patients show ventilator dependence and need prolonged ventilator support.

### Prognosis and outcome [4, 5, 10, 11]

Overall, the prognosis is good (with paraneoplastic anti-NMDAR encephalitis having a better prognosis than most other paraneoplastic encephalitides and in those with an associated malignancy than those without any association), with nearly half the patients making a full recovery and a quarter of them having only mild residual deficits. However, 25% of patients may have severe residual deficits or eventually succumb to the disease process. The outcome is better for those who undergo early tumor removal, especially if this is done within four months of the onset of symptoms.

The importance of tumor excision is also suggested, as patients who do not undergo excision develop recurrent neurological symptoms heralding a tumor recurrence. Further, dramatic recovery can occur in some patients, as was seen in our patient, suggesting a potentially reversible neuronal dysfunction. But, without surgical intervention or with late tumor removal, the clinical course can be prolonged or even fatal, with a mortality rate of around 4%. The usual causes include sepsis, cardiac arrest, acute respiratory distress, refractory status epilepticus or secondary tumor progression.

Recovery occurs in the reverse order of symptom presentation with autonomic and respiratory functions being the first to recover, followed by the interactive and speech functions. Social behaviour and executive function symptoms are usually the last to improve.

## Conclusions

A paraneoplastic syndrome with an ovarian tumor should always be in the differential diagnosis in a young woman with new-onset psychiatric symptoms. The clinical improvement in these patients is slow even after tumor excision. Early use of corticosteroids and IVIG may improve the outcome, and early tumor removal improves the prognosis.

## Consent

Written informed consent was obtained from the patient for publication of this case report and any accompanying images. A copy of the written consent is available for review by the Editor-in-Chief of this journal.

## Abbreviations

ANA: anti-nuclear antibody
ANCA: anti-nuclear cytoplasmic antibody
APLA: anti-phospholipid antibody
CSF: cerebrospinal fluid
EEG: electroencephalogram
FLAIR: fluid attenuated inversion recovery
HIV: human immunodeficiency virus
IVIG: intravenous immunoglobulin
IVIg: Intravenous immunoglobulin
MRI: magnetic resonance imaging
NMDA: N-methyl D-aspartate
NMDAR: NMDA receptor
PLE: paraneoplastic limbic encephalitis
T2W: T2 weighted images.
